# Differential prognostic impact of stratified additional chromosome abnormalities on disease progression among Malaysian chronic myeloid leukemia patients undergoing treatment with imatinib mesylate

**DOI:** 10.3389/fonc.2022.720845

**Published:** 2022-08-08

**Authors:** Ismail Siti Mariam, Ramli Norhidayah, Abu Bakar Zulaikha, Mohd Yunus Nazihah, Hassan Rosline, Ghazali Anis Kausar, Sulong Sarina, Husin Azlan, Ravindran Ankathil

**Affiliations:** ^1^ Human Genome Centre, School of Medical Sciences, Universiti Sains Malaysia, Kubang Kerian, Kelantan, Malaysia; ^2^ Department of Haematology, School of Medical Sciences, Universiti Sains Malaysia, Kubang Kerian, Kelantan, Malaysia; ^3^ Unit of Biostatstics and Research Methodology, School of Medical Sciences, Universiti Sains Malaysia, Kubang Kerian, Kelantan, Malaysia; ^4^ Internal Medicine, School of Medical Sciences, Universiti Sains Malaysia, Kubang Kerian, Kelantan, Malaysia

**Keywords:** chronic myeloid leukemia, additional chromosome abnormalities (ACAs), disease progression, imatinib mesylate, resistance, stratification

## Abstract

The emergence of additional chromosome abnormalities (ACAs) in chronic myeloid leukemia (CML) patients during treatment with a tyrosine kinase inhibitor (TKI) regime is generally associated with resistance to treatment and a sign of disease progression to accelerated phase or blast phase. We report the type, frequency, and differential prognostic impact of stratified ACAs with treatment response in 251 Malaysian CML patients undergoing TKI therapy. ACAs were observed in 40 patients (15.9%) of which 7 patients (17.5%) showed ACAs at time of initial diagnosis whereas 33 patients (82.5%) showed ACAs during the course of IM treatment. In order to assess the prognostic significance, we stratified the CML patients with ACAs into four groups, group 1 (+8/+Ph), group 2 (hypodiploidy), group 3 (structural/complex abnormalities); group 4 (high-risk complex abnormalities), and followed up the disease outcome of patients. Group 1 and group 2 relatively showed good prognosis while patients in group 3 and group 4 had progressed or transformed to AP or blast phase with a median survival rate of 12 months after progression. Novel ACAs consisting of rearrangements involving chromosome 11 and chromosome 12 were found to lead to myeloid BP while ACAs involving the deletion of 7q or monosomy 7 led toward a lymphoid blast phase. There was no evidence of group 2 abnormalities (hypodiploidy) contributing to disease progression. Compared to group 1 abnormalities, CML patients with group 3 and group 4 abnormalities showed a higher risk for disease progression. We conclude that the stratification based on individual ACAs has a differential prognostic impact and might be a potential novel risk predictive system to prognosticate and guide the treatment of CML patients at diagnosis and during treatment.

## Introduction

Chronic myeloid leukemia (CML) is a myeloproliferative disorder characterized by the presence of a cytogenetic marker known as the Philadelphia chromosome, resulting from the reciprocal translocation between chromosomes 9 and 22 t(9;22) (q34;q11). This translocation leads to the generation of a *BCR::ABL1* chimeric gene that results from the fusion of the *ABL1* gene on chromosome 9 with the *BCR* gene on chromosome 22. The *BCR::ABL1* fusion gene is an oncogene which encodes a constitutively activated tyrosine kinase protein with a molecular weight of p190, p210, or p230 kDa depending on the breakpoint located within the *BCR* gene that is fused together with *ABL1* on exon a2 to form subtypes e1a2, e19a2, and e14a2 respectively. This oncogenic tyrosine kinase protein is responsible for the leukemic phenotype, through the constitutive activation of multiple signaling pathways (1). At diagnosis, most of the CML patients show the classical t(9;22) (q34;q11) translocation or its variant, as the sole abnormality. However, in a small proportion of CML cases, additional chromosome abnormalities (ACAs) in Ph+ cells may appear. ACAs are chromosomal abnormalities observed, in addition to the Ph′ chromosome in CML patients, seen at the time of diagnosis or during the course of treatment.

In the era of tyrosine kinase inhibitor (TKI) therapy, most patients with chronic-phase (CP) CML can have normal-life expectancy (2). However, ~5% of patients will progress to blast transformation with an annual incidence of 1% to 1.5% (3). Although TKI may exhibit some degree of activity in the blast phase (BP), the rate and duration of response are less favorable than in patients whose disease is in CP and can lead to resistance and relapse (4–6). Intensive chemotherapy in BP can be effective but is less successful than in *de novo* acute leukemia. Overall, the median survival in patients with BP-CML is usually less than 1 year, even in the TKI era (5, 7–11). This high fatality of blast-phase patients warrants the need for early identification of high-risk patients who are likely to progress to blast phase and thus to prevent the onset of BP.

Even though the molecularly targeted TKI drug Imatinib Mesylate (IM) has become the gold standard drug for the treatment of chronic myeloid leukemia (CML), development of resistance in nearly 20%–35% of CML patients on IM therapy is a daunting problem (12). Resistance to IM could be due to a heterogeneous array of factors. Besides mutation and amplification of the *BCR::ABL1* gene being the major risk factors for resistance, pharmacokinetic variability as a result of genetic polymorphisms in pharmacogenes related to IM binding, metabolism, and transport may also influence the intracellular delivery, bioavailability, and effectiveness of IM and hence modulate the clinical response to IM. The various mechanisms of resistance do not fall within the scope of this article.

In CML, the emergence of additional chromosome abnormalities (ACAs) subsequent to the initial t(9;22) translocation or during the course of treatment is considered as a hallmark of multistep disease progression. The appearance of ACAs during IM treatment commonly known as clonal evolution (CE) has also been reported to play an important role in IM resistance (13, 14).

Based on their frequency, ACAs had been earlier stratified into major (trisomy 8, i(17q), trisomy 19, and extra copy of the Ph chromosome) and minor route abnormalities (15). However, the stratification of ACAs into major and minor route does not necessarily reflect the underlying biology or their impact on disease progression. According to Arber etal. (16), major-route ACAs at initial diagnosis and any ACAs in Ph^+^ cells acquired during therapy are considered as a criteria for defining AP (16). Additional chromosomal abnormalities in Ph chromosome-positive cells are important in the determination of disease progression and patient’s outcome. Even though the association between major-/minor-route ACAs is known, not many reports are available on the differential prognostic impact of individual ACAs on disease progression. This study aimed to investigate the ACAs observed in Malaysian CML patients at the time of initial diagnosis as well as during the course of IM treatment and to determine the differential prognostic impact of ACAs on disease progression.

## Methodology

### Patient selection

This study has approval from the Human Research and Ethics Committee of USM (USM/JEPeM/18110664–14/02/2019) and Ministry of Health (MOH) Malaysia. A total of 251 CML patients were recruited based on two criteria: a) CML patients who were presented with t(9;22)(q34;q11) or a variant Ph chromosome, detected by conventional cytogenetic analysis and confirmed by fluorescence *in situ* hybridization (FISH) and/or molecular testing for *BCR::ABL1* fusion transcripts, and b) CML patients who were treated orally with 400/600 mg of IM as frontline treatment for at least 12 months. Chronic-phase patients received first-line treatment with imatinib 400 mg orally once daily whereas accelerated-phase patients received 600 mg once daily as per European Leukemia Net guidelines (17). We excluded atypical CML cases, CML patients who were previously on other type of treatment and who had undergone bone marrow transplant. The patients were recruited from Hospital Universiti Sains Malaysia (HUSM) and various other hospitals under the Ministry of Health Malaysia, such as Hospital Ampang, Hospital Pulau Pinang, and Hospital Umum Sarawak.

### Karyotype analysis

Fresh bone marrow aspirate of the recruited CML patients was cultured overnight in RPMI 1640 medium at 37˚C and 5% CO_2_. Synchronized cell cultures were carried out, and the GTG-banded metaphases prepared were analyzed using an automated karyotyper (Cytovision 4.2 and MetaSystems Software) (18). A minimum of 20 GTG-banded metaphases were analyzed for each sample. The clonality of ACAs present in Ph-positive CML patients at the diagnosis stage or during treatment with TKI and also clonal chromosome abnormalities in patients who achieved a Ph-negative status as a result of a complete cytogenetic response (CCyR) were defined according to the International System for Human Cytogenomic Nomenclature (19).

### Assessment of response/resistance

#### Hematological response

A complete hematological response was recorded when there was normalization of peripheral blood counts with leukocyte count <10 × 10^9^/l, platelet count <450 × 10^9^/l, and no immature cells in peripheral blood. The assessment of CHR is one of the first steps in measuring treatment efficacy in CML patients (20).

#### Cytogenetic response

Cytogenetic response (CyR) was evaluated at month 6 of the TKI treatment, every 6 months until CCyR response was achieved, every 12 months since CCyR, and at any time on clinical suspicion of progression as per European Leukemia Net (ELN) guidelines (21). An optimum response was recorded when there was <35% of Ph+ chromosome at 3 months and no Ph+ at 6 months. Failure was denoted when there was presence of >95% Ph+ in 3 months, >35% Ph+ in 6 months, and >1% in 12 months after TKI treatment. Loss of cytogenetic response at any time was considered as treatment failure. Both molecular and cytogenetic tests are recommended particularly in cases where the response is borderline or fluctuating (22).

Based on the type and frequencies of ACAs encountered in CML patients, they were categorized into four groups following Gong et al.’s (23) four-tier risk stratification valid for ACAs detected at diagnosis of CML with slight modifications. Group 1 comprised the presence of either trisomy 8 or double Ph, and group 2 comprised the presence of a hypodiploid karyotype. Karyotypes with structural abnormalities were categorized under group 3, and high-risk complex karyotypes were categorized under group 4. These patients were followed up for a period of 6–171 months with the median of 54.5 months to evaluate their prognostic significance. For assessing the prognostic risk of ACAs, Ph-positive CML patients with ACAs and Ph-negative CML patients ACAs were grouped separately and analyzed, following Issa etal. (24).

#### Statistical analysis

A univariate analysis was carried out to assess the risk of clinical parameters at diagnosis and the stratified ACA subgroups with disease progression. A multivariate analysis for risk of disease progression could not be carried out as some of the clinical parameters were not uniformly available for some of the patients during the course of treatment. The Kaplan–Meier method was used to analyze the overall survival (OS), and the differences in OS between the four groups (group 1, group 2, group 3, and group 4) were evaluated by the log-rank test using IBM SPSS statistics 26 software. The OS was calculated from two different start points: (1) date of initial CML diagnosis and (2) date of emergence of first ACAs. The end point was the date of last follow-up or death.

## Results

**Figure 1:**
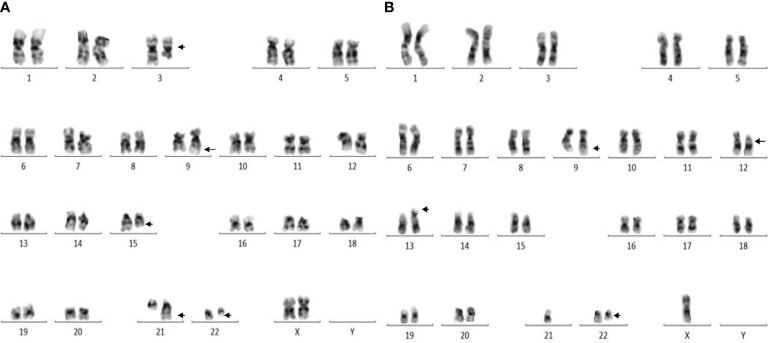
**(A)** Complex karyotype (patient #26) showing 46,XX,t(9;22)(q34;q11),t(3;15;21)(q25;q22;q22) pattern. **(B)** Complex karyotype (patient # 37) showing 44,X,-Y,t(9;22)(q34;q11),del(12)(p12),der(13;21)(q10;q10) pattern.

A total of 251 CML patients were included in this study (consisting of 130 men and 121 women) aged between 15 and 86 years with a median age of 42.0 years. ACAs were encountered in 40 patients (15.9%), of which seven (17.5%) were observed in patients who showed complete cytogenetic response and attained a Ph-negative status whereas in the remaining 33 patients (82.5%) ACAs were observed when the patients were still Ph positive. The ACAs in this study were seen almost in equal proportions among male (45.5%) and female (54.5%) patients. Among these 33 patients, 22 (66.7%) were in CP, six (18.2%) were in AP, and five (15.2%) were in BP at the diagnosis stage. Out of these 33 patients, eight (24.2%) had shown additional abnormalities at the time of initial diagnosis while the remaining 25 patients (75.8%) had shown ACAs during IM treatment. Out of these eight patients with ACAs at time of diagnosis, six are still alive and two had deceased. Follow-up of these 33 patients with ACAs showed that 20 (50.0%) had progressed to accelerated or blast phase, of which 15 patients (37.5%) had transformed to myeloid blast phase, three (7.5%) to lymphoid blast phase, and another two (5.0%) to accelerated phase with blast counted less than 10% in bone marrow or peripheral blood. Nine patients (22.5%) in the group 1 with presence of trisomy 8 and double Ph (**Table 1A**) and another nine (22.5%) in the group 2 had hypodiploid karyotype (**Table 1B**). Among 40 patients, 31 patients (77.5%) had complex karyotypes involving structural abnormalities (**Figures 1A, B**).

**Table 1:** (A): Karyotype pattern, disease progression, and outcome of four groups of CML patients with ACAs.

Patient/sex/age at diagnosis	Karyotype	Spleen size(cm)	% blast in PB	Platelet count(×10^3^/µL)	Disease progression	CyR	Interval* in months	Last status
1(F/33)	46,XX,t(9;22)(q34;q11)[20]/47,XX,+8[2]	19	No	323	No	No	46	Alive
2(M/30)	46,XY,t(9;22)(q34;q11)[17]/47,XY,idem,+der(22)t(9;22)[2]/46,XX[6]	24	2	463	No	No	90	Alive
3(F/39)	46,XX,t(9;22)(q34;q11)[16]/47,XX,+8[3]/46,idem,del(5)(q31),del(13)(q31)[2]/46,XX[2]	14	No	227	No	No	26	Alive
4(F/40)	46,XX,t(9;22)(q34;q11) [8]/47,XX,+8[7]/46,XX[2]	11	No	467	Myeloid blast	No	12	Alive
5(F/43)	47,XX,+8[5]	14	No	444	No	Complete	14	Alive
6(M/49)	46,XY,t(9;22)(q34;q11)[20]/47,idem,+der(22)t(9;22)[11]	12	No	553	Myeloid blast	No	20	Dead
7(M/41)	47,XY,+8,t(9;22)(q34;q11)[18]/	25	No	648	No	No	7	Alive
8(F/41)	46,XX,t(9;22)(q34;q11)[12]/47,XX,+8 [3]	20	No	893	No	No	37	Alive
9(F/21)	46,XX,t(9;22)(q34;q11)[26]/92,XXXX,t(9;22)(q34;q11)x2[4]	26	27	440	Myeloid blast	No	0	Alive

*Interval (month) between CML diagnosis and emergence of ACAs.

PB, peripheral blood; CyR, cytogenetic response. **Group 1** (Trisomy 8 and double Philadelphia chromosome).

**Table 1:** (B): Group 2 (Hypodiploidy).

Patient/sex/age at diagnosis	Karyotype	Spleen size(cm)	% blast in PB	Platelet count(×10^3^/µL)	Disease progression	CyR	Interval* in months	Last status
10(M/57)	45,XY,-18[3]/46,XY[15]	2	No	610	No	Complete	29	Alive
11(M/36)	38~45,XY,-16[3], -18[5],-21[4][cp13]/46,XY[22]	12	No	359	No	Complete	100	Alive
12(F/43)	45,XX,-10[10]/46,XX[3]	20	6	584	No	Complete	28	Alive
13(F/49)	46,XX,t(9;22)(q34;q11)[12]/ 37 ~ 45,idem, -8[3], -19[3], -20[7],-22[3][cp11]	23	No	1062	No	No	0	Alive
14(M/20)	41~45,XY,-4[3],-22[3] [cp10]/46,XY,t(9;22)(q34;q11)[1]/46,XY[19]	23	2	706	No	Complete	6	Alive
15(M/21)	41~45,XY,-8[3],-15[4],-19[4][cp6]/46,XY,t(9;22)(q34;q11)[1]	21	17	705	No	No	0	Alive
16(M/22)	44~45,XY,-16[3],-21[4][cp6]/46,XY[20]	18	2	109	No	Complete	3	Alive
17(F/54)	41 ~45,XX,-4[3],-15[3] [cp8]/46,XX[20]	16	No	271	No	Complete	84	Alive
18(M/18)	46,XY,t(9;22)(q34;q11)[25]/42~45,X,-Y[3], -21[3] [cp13]/46,XY[2]	22	No	321	No	No	101	Alive

*Interval (month) between CML diagnosis and emergence of ACAs.

PB, peripheral blood; CyR, cytogenetic response.

With regard to the types of abnormalities in these 33 patients, three (9.1%) showed monosomy 7/del (7q) in group 4 (patients #29, #36, and #38 in **Table 1D**) and three (9.1%) (patients #19 and #25 in group 3 and #30 in group 4) showed structural chromosome rearrangements involving chromosomes 1, 2, and 11 (**Tables 1C**, **D**). Among these three patients, patient #30 showed der(11) as a result of translocation of segment p14 of chromosome 2 to chromosome 11 at p15 and patient #25 showed a translocation between chromosomes 1 and 11 involving segments 1q22 and 11p12. The third patient (patient #19 in **Table 1C**) showed a deletion of a short arm of chromosome 11 at p13. Another three (9.1%) patients showed structural abnormalities involving chromosomes 9, 12, and 17, as shown in **Tables 1C**, **D**. Patient #23 (**Table 1C**) showed der(9) as a result of translocation between chromosomes 9 and 12 involving 9p24 and 12p13 segments, and patient #21 (**Table 1C**) showed translocation between chromosomes 12 and 17 involving 12p13 and 17q21 segments. Two of our patients (patient #26 and #31) showed abnormalities involving chromosome 15. Patient #26 (**Table 1C**) showed a three-way translocation involving chromosomes 3, 15, and 21 with rearrangement of breakpoints at 3q25 of chromosome 3, 15q22 of chromosome 15, and 21q22 of chromosome 21. The other patient (#31 **Table 1D**) showed a deletion of chromosome 15 at q24 and an additional subclone involving translocation t(3;15) (q21;q15). Complex abnormalities involving i(17)(q10) along with trisomy 8 and/or double Ph chromosome were observed in three patients. Yet another three patients showed addition of trisomy 19 along with trisomy 8 or additional der(22) t(9;22). All these abnormalities are shown in **Table 1D** (patients #33, #34, and #40).

**Table 1:** (C): Group 3 (structural abnormalities involving chromosomes 1, 3, 6, 9, 10, 11, 12, and 17).

Patient/sex/age at diagnosis	Karyotype	Spleen size(cm)	% blast in PB	Platelet count(×10^3^/µL)	Disease progression	CyR	Interval* in months	Last status
19(F/32)	46,XX,t(9;22)(q34;q11),del(11)(p13)[17]	17	4	221	Myeloid blast	No	32	Dead
20(F/24)	46,XX,t(9;22)(q34;q11)[5]/46,XX,+1,del(1)(p13),der(7)t(1;7)(q31;p22),del(9)(q13),t(9;22)(q34;q11),-19[2]	24	40	594	No	No	19	Alive
21(F/50)	46,XX,t(9:22)(q34;q11)[9]/46,idem,t(12;17)(p13;q21) [6]	23	5	1477	AP	No	42	Dead
22(F/52)	46,XX,t(9;22)(q34;q11)[20]/46,idem,ins(10)(p15q25q26)[2]/46,XX[3]	4	No	101	AP	No	13	Dead
23(F/46)	46,XX,t(9;22)(q34;q11)[2]/46,XX,t(9;22)(q34;q11),der(9)t(9;12)t(p24;p13)[15]	10	No	112	Myeloid blast	No	9	Dead
24(F/37)	46,XX,del(1)(p32) [11]/46,idem,t(9;22)(q34;q11)[2]	13	No	457	Myeloid blast	No	83	Dead
25(M/18)	46,XY,t(1;11)(q22;p12),t(9;22)(q34;q11)[23]	27	No	331	No	Complete	0	Alive
26(F/63)	46,XX,t(9;22)(q34;q11)[8]/46,idem,t(3;15;21)(q25;q22;q22) [13]	6	No	132	Myeloid blast	No	0	Dead
27(M/57)	46,XY,del(3)(q27)[15]	11	2	436	No	Complete	8	Alive
28(M/20)	43~46,XY,del(6)(p21)[4],t(9;22)(q34;q11)[4], -19[4],+22[3][cp4]	25	No	611	No	No	0	Alive

*Interval (month) between CML diagnosis and emergence of ACAs.

PB, peripheral blood; CyR, cytogenetic response; AP, accelerated phase.

**Table 1:** (D): Group 4 (complex karyotype).

Patient/sex/age at diagnosis	Karyotype	Spleen size(cm)	% blast in PB	Platelet count(×10^3^/µL)	Disease progression	CyR	Interval* in months	Last status
29(F/19)	43~45,XX, t(9;22)(q34;q11),-10,-22[cp3]/45,XX,-7,i(9)(q10),t(9;22)(q34;q11)[3]/46,XX[2]	25	5	240	Lymphoid blast	No	6	Alive
30(M/59)	46,XY,t(9;22)(q34;q11),der(11)t(2;11)(p14;p15)[1]/46,sl,der(13)t(13;17)(p11;p12)[14]/47,sdl1,+der(22)t(9;22)[5]/48,sdl2,+8[4]/47,sdl1,+8[1]	13	No	470	Myeloid blast	No	21	Dead
31(M/54)	46,XY,t(9;22)(q34;q11)[20]/46,idem,del(15)(q24)[2]/46,idem,t(3;15)(q21;q15),del(15)(q24),del(17)(q22)[2]/47,idem,+8 [1]	13	10	962	Myeloid blast	No	26	Alive
32(F/14)	46,XX,t(9;22)(q34;q11)[2]/49,idem,+8,+13,+19[18]	21	14	480	Myeloid blast	No	10	Dead
33(F/54)	46,XX,t(9;22)(q34;q11)[10]/48,idem, +8, i(17)(q10),+der(22)t(9;22) [17]	13	No	542	Myeloid blast	No	22	Dead
34(M/60)	48,XY, +8,t(9;22)(q34;q11),+der(22)t(9;22)[3]/48,XY,t(9;22)(q34;q11),+19,+der(22)t(9;22) [2]/49,XY,+8,t(9;22)(q34;q11),+19,+der(22)t(9;22) [18]	10	No	1759	Myeloid blast	No	14	Dead
35(F/49)	47,XX,t(9;22)(q34;q11), i(17)(q10),+der(22)t(9;22)[9]	12	No	153	Myeloid blast	No	34	Dead
36(M/20)	45,XY,-7,der(9)t(7;9;22)(q11;q34;q11.2)[15]/46,idem,+8[7]	5	No	411	Lymphoid blast	No	7	Dead
37(M/39)	45,X,-Y,t(9;22)(q34;q11)[27]/44,idem,-20[4]/38~44,idem,-8[3],-14[4],-16[4],-18[3][cp13]/44,idem,del(12)(p12),der(13;21)(q10;q10)[3]/44,idem,-13,-19,+der(22)t(9;22)[1]	11	No	500	Myeloid blast	No	6	Dead
38(M/40)	46,XY,t(9;22)(q34;q11)[14]/39 ~ 45,idem,-6[3],?del(7)(q33)[2],-18[3],-21[3][cp13]/46,XY[1]	23	>20	93	Lymphoid blast	No	0	Dead
39(F/27)	46,XX,t(4;9;22)(q32;q34;q11)[18]/47,idem,+19[15]/46,XX[1]	24	No	1200	Myeloid blast	No	11	Dead
40(M/32)	46,XY,t(9;22)(q34;q11)[22]/47 ~ 50,idem, +8[2],i(17)(q10)[3], +19[2] [cp3]	24	No	201	No	Complete	0	Alive

*Interval (month) between CML diagnosis and emergence of ACAs.

PB, peripheral blood; CyR, cytogenetic response.

To determine the differential prognostic significance of ACAs, these 40 patients were divided into four groups based on the type of individual ACAs observed, as described previously. Trisomy 8 or double Ph chromosome were included in group 1. Group 2 included hypodiploid karyotypes where the commonly missing chromosomes were monosomies of chromosomes 4, 8, 15, 16, 18, 19, 21, and 22 and loss of the Y chromosome. Group 3 abnormalities comprised single or multiple structural rearrangements predominantly involving chromosomes 1, 3, 6, 9, 10, 11, 12, and 17. Group 4 included complex karyotypes with abnormalities involving a combination of three or more chromosomes such as tetrasomy 8, trisomy 19, iso(17q), and double Ph chromosome as ACAs.

**Figure 2:**
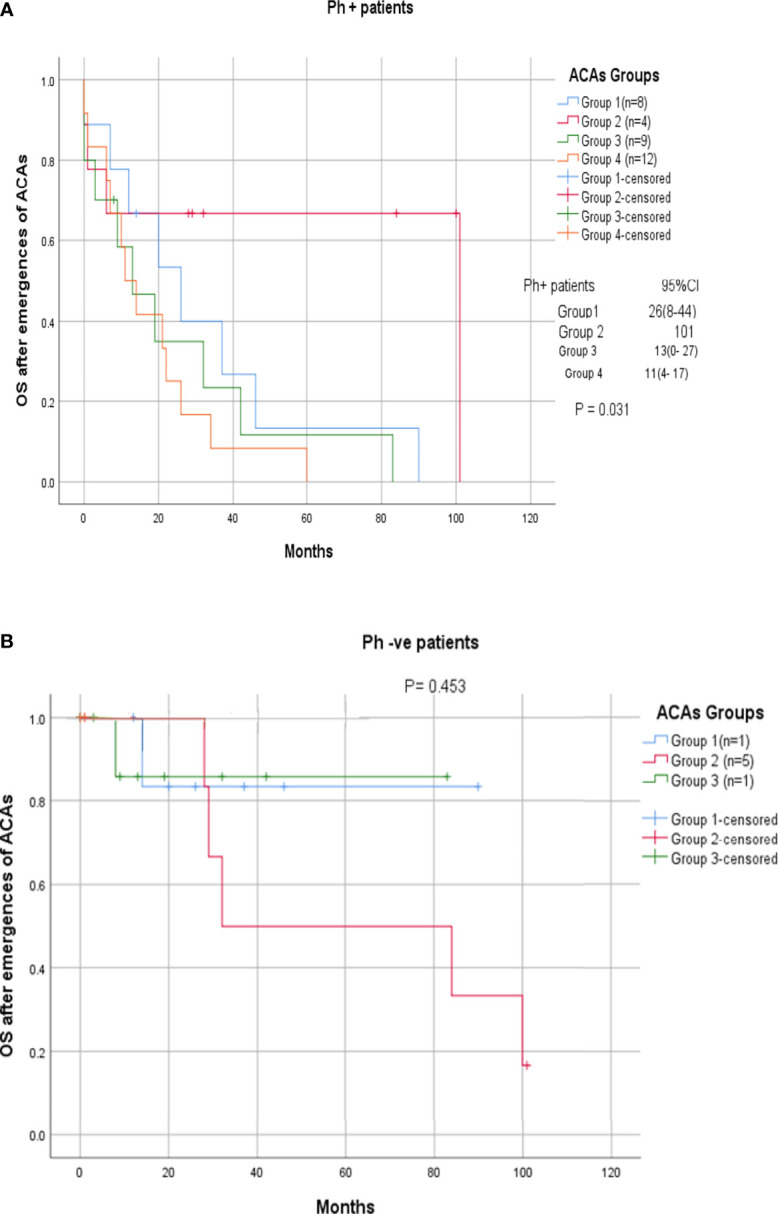
**(A, B)** CML patients with different groups of ACAs (group 1, group 2, group 3, and group 4) and their overall survival rate.

**Figure 3:**
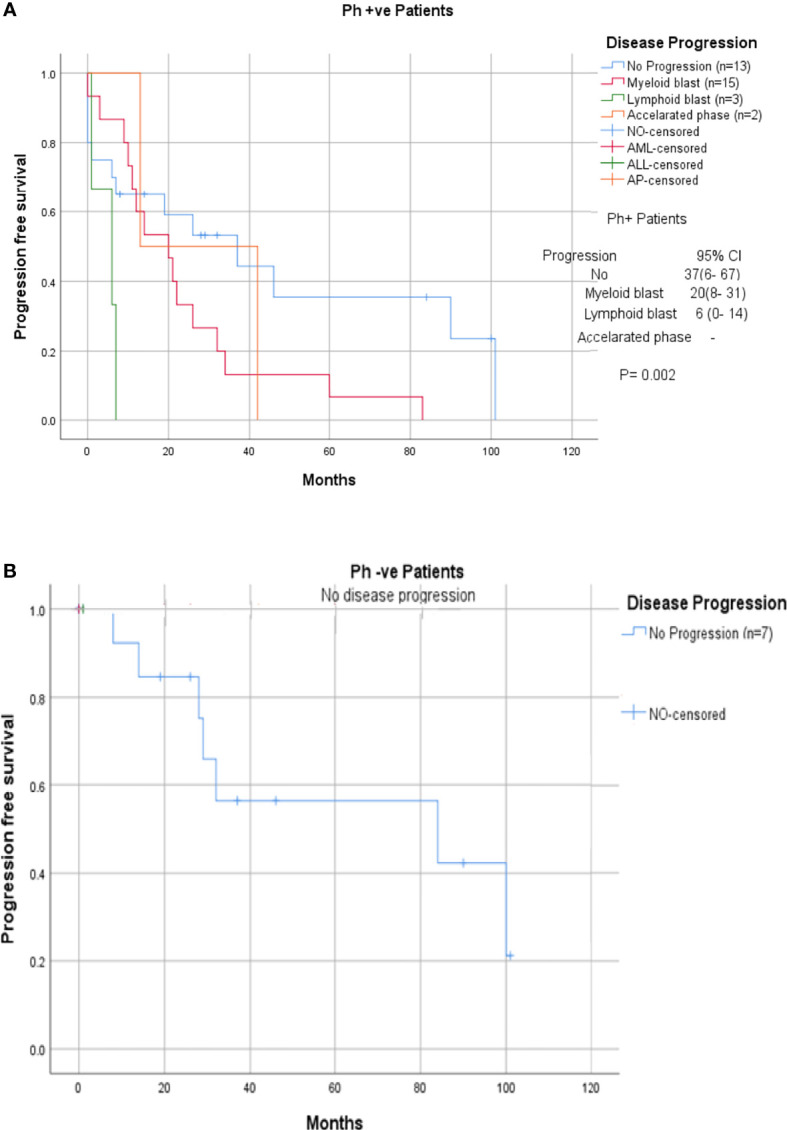
**(A, B)** CML patients with different groups of ACAs (group 1, group 2, group 3, and group 4) and their respective of progression-free survival.

Among the 33 Ph-positive patients with ACAs, 26 patients (78.8%) showed no cytogenetic response, four (12.1%) encountered loss of cytogenetic response whereas three (9.1%) achieved a complete cytogenetic response. During the follow-up period of 171 months of these 33 patients with ACAs, 13 (39.4%) did not enter into disease progression and remain in the chronic phase. In the remaining 20 patients, it was observed that 18 (54.5%) had transformed to blast phase and another two (6.1%) had progressed to accelerated phase. Among the 18 patients who progressed to blast phase, 15 (45.5%) had transformed to myeloid blast phase and three (9.1%) had transformed to lymphoid blast phase. Patients in groups 3 and 4 had poor outcome with occurrence of disease progression within 14 to 32 months from the emergence of ACAs and deceased within 6 to 12 months. Out of the total 33 Ph-positive patients with ACAs, 15 (45.5%) patients transformed to myeloid blast phase and another 3 (9.1%) transformed to lymphoid blast phase with median survival rates less than 1 year (**Table 2**). Sixteen (48.5%) patients with structural abnormalities showed poor survival when compared with the patients who showed numerical abnormalities of either trisomy or hypodiploid (**Figure 3A**). However, three patients in group 1 with trisomy 8 or double Ph had transformed to myeloid blast phase but showed good prognosis (**Figure 3A**). When the association of various clinical parameters at diagnosis (age, gender, spleen size, and platelet count) with disease progression was assessed (**Table 3**), none of these emerged as having significant impact. Among all patients, 16 (40%) had passed away of which 8 (50%) was due to septicemia shock, four (25%) due to intracranial bleeding from leukemia infiltration, and another four (25%) due to other causes (**Table 2**). For determining the risk association of the four stratified groups of ACAs with disease progression, Ph-positive patients with ACAs (33) and Ph-negative patients with ACAs (7) were analyzed separately. **Table 4** shows the risk association of the four groups of ACAs with disease progression in Ph-positive and Ph-negative cases. In the Ph-negative CML patients with ACAs, the differential risk of stratified ACAs on disease progression could not be carried out because none of the patients in this group developed disease progression (**Figures 2B**, **3B**). In the group of Ph-positive CML patients with ACAs, those patients harboring group 3 and group 4 abnormalities showed significantly highest risk for disease progression. For group 2, the differential risk could not be assessed as none of them showed disease progression. **Figures 2A**, **3A** represent the progression -free survival and overall survival rates in Ph- positive patients and **Figure 2B**, **3B** in the Ph- negative patients.

**Table 2:** Clinical characteristics, disease progression, and prognosis of four groups of CML patients with ACAs at diagnosis and during treatment.

	Group 1 n = 9	Group 2 n = 9	Group 3 n = 10	Group 4 n = 12
**Patients**				
Ph+ with ACAs Ph- with CCA	8 (88.9%) 1 (11.1%)	4 (44.4%) 5 (55.6%)	9 (90%) 1 (10%)	12 (100%) 0
**Sex**				
Male Female	3 (33.3%) 6 (66.7%)	6 (66.7%) 3 (33.3%)	3 (30.0%) 7 (70.0%)	7 (58.3%) 5 (41.7%)
**Age at Dx,y**				
Median Range	44 25–60	42 15–60	57 21–65	57 21–65
*Interval 1, mo				
Median Range	NR	NR	32 0–70.5	14 2.1–25.9
*Interval 2, mo				
Median Range	NR	NR	12 4.0–20.0	6.0 0.5–11.5
**Emergence of ACAs**				
Diagnosis After treatment	1 (11.1%) 8 (88.9%)	2 (22.2%) 7 (77.8%)	3 (30%) 7 (70%)	2 (16.7%) 10 (83.3%)
**Disease progression**				
No AP BP	6 (66.7%) 0 3 (33.3%)	9 (100%) 0 0	4 (40%) 2 (20%) 4 (40%)	1 (8.3%) 0 11 (91.7%)
**BP phenotype**				
Myeloid Lymphoid	3 (100%) 0	0 0	4 (100%) 0	8 (72.7%) 3 (27.3%)
**Cytogenetic response**				
CCyR No CyR Loss CyR	1 (11.1%) 8 (88.9%) 0	5 (55.6%) 2 (22.2%) 2 (22.2%)	2 (20%) 7 (70%) 1 (10%)	1 (8.3%) 10 (83.4%) 1 (8.3%)
**Status of last follow-up**				
Alive Dead	8 (88.9%) 1 (11.1%)	9 (100%) 0	4 (40%) 6 (60%)	3 (25%) 9 (75%)
**Allo-SCT**	1	1	0	0

*INTERVAL 1—from emergence of ACAs to disease progression.

*INTERVAL 2—disease progression to last follow up/death.

NR, not reached; M/o, months; ACAs, additional chromosome abnormalities; CCA, clonal chromosome abnormalities.

**Table 3:** Risk association of clinical parameters at diagnosis with the disease progression in CML patients.

Clinical characteristic		OR (95% CI)	P-value	Adjusted OR (95% CI)	P-value
Age		0.16 (0.987–1.083)	0.16		
Gender	Male	1 (reference)		1 (reference)	
	Female	2.79 (0.77–10.04)	0.12	3.79 (0.74–19.53)	0.13*
Spleen size	<12 cm	1 (reference)		1 (reference)	
	12–20 cm	0.28 (0.04–1.89)	0.19	0.22 (0.02–2.07)	0.054*
	>20 cm	0.16 (0.02–1.001)	0.05	0.11 (0.12–1.04)	0.054*
Platelet count	150–450 × 10^3^/µL	1 (reference)		1 (reference)	
	<150 × 10^3^/µL	7.20 (0.62–83.34)	0.11	11.26 (0.49–259.23)	0.13*
	>450 × 10^3^/µL	1.98 (0.49–7.94)	0.34	2.65 (0.56–12.65)	0.22*

*Statistically not significant (P > 0.05).

**Table 4:** Risk association of the four stratified groups of ACAs with disease progression among Ph+ positive and Ph- negative CML patients.

ACAs groups	Progression (n = 34)%	No progression (n = 215)%	OR (95% CI)	P-value
**No ACAs**	14 (41.2)	195 (90.8)	1 (reference)	
**Ph^+^-positive CML with ACAs**				
Group 1	3 (8.8)	5 (2.8)	8.61 (1.86–39.80)	0.006*
Group 2	0 (0.0)	4 (1.0)	N/A	N/A
Group 3	6 (17.6)	3 (1.4)	28.71 (6.48–127.16)	0.001*
Group 4	11 (32.4)	1 (0.5)	157.93 (19.00–1312.58)	0.001*
**Ph^–^negative CML with ACAs**				
Group 1	0 (0.0)	1 (0.5)	N/A	N/A
Group 2	0 (0.0)	5 (2.3)	N/A	N/A
Group 3	0 (0.0)	1 (0.5)	N/A	N/A
Group 4	0 (0.0)	0 (0.0)	N/A	N/A

Bold is statistically significant (p<0.05).

N/A - Not applicable.

## Discussion

Definite protocols for evaluating the response of CML patients to treatment have been developed during the TKI era. Accordingly, monitoring of CML patients on TKI treatment for emergence of any ACAs, both at the time of diagnosis and during the course of treatment, is very important. This could be indicative of patients’ transformation to advance stage (accelerated or blast) or treatment failure. In the present study, we analyzed the types and frequencies of ACAs present at diagnosis and during the course of IM treatment in CML patients who were Ph positive and who became Ph negative as a result of CCyR to IM treatment and the differential impact of stratified ACAs on treatment response and disease progression. To the best available knowledge, this is the first and one of the largest studies describing the differential prognostic impact of stratified ACAs in 251 Malaysian CML patients undergoing imatinib treatment.

Although in CML, a male predominance had been documented, our patients showed almost an equal proportion of male and female (1.1:1). This finding is contradictory with other reports (25) in which the occurrence of CML was reported to be slightly higher in men than in women. It is presumed that Malaysian women may be having almost the same degree of risk for CML susceptibility as Malaysian men. However, further studies involving a higher sample size are warranted to confirm this finding. The median age of Malaysian CML patients was 42 years. This is a little bit early age of onset of the disease compared to a median age of 57 years for CML patients in the Western population (26). Nevertheless, our finding is in concordance with Au etal. (27) who reported that the occurrence of CML in Asia Pacific countries tends to afflict the younger population. When the impact of other clinical parameters at diagnosis on disease progression was assessed, none of them emerged as having a significant impact. This is not in agreement with Racil etal. (28) and other researchers (29, 30) who reported that enlarged spleen correlated with grade of leukocytosis and also with progression of CML to advanced stages and with the prognosis of CML patients. In our study, the presence of ACAs was predominantly in younger patients with low incidence of mortality compared to older-age patients. Resistance to IM had been reported to be higher in advance-phase patients who presented with ACAs (clonal evolution) in a Philippine population (27). Lu (31) had reported a primary resistance rate of 16% in AP and an increased rate up to 45% in blast phase in a Chinese population, due to drug resistance. Patients with disease progression to advanced stage are switched to the second generation of TKIs (32).

Resistance to IM and disease progression had been reported to be due to evolution of the disease with occurrence of additional numerical or structural chromosomal aberrations that lead to *BCR::ABL1-*independent proliferation of leukemic cells (33). The emergence of ACAs during disease progression has been implicated to be due to the environment of genomic instability (34). Enhanced DNA damage from accumulation of reactive oxygen species followed by compromised DNA repair due to long-standing BCR-ABL1 expression has been reported to lead to genomic instability (14). However, the precise etiology of these ACAs or the pathologic link between these abnormalities and CML disease progression has not been fully elucidated.

Clonal cytogenetic evolution with ACAs has been linked with decreased response to the TKI group and worse survival. Despite ACAs being considered as a sign of disease progression, the differential prognostic impact of individual ACAs remains unclear. So far, there is no classification system for a differential prognostic indication of specific ACAs in CML patients undergoing treatment with TKIs. The European Leukemia Net recommendations (2013) for the management of CML considers major-route but not minor-route ACAs, emerging during therapy as defining criteria for AP (21). Meanwhile, all ACAs emerging during therapy are considered defining criteria for AP by the 2016 update of WHO classification of tumors of hematopoietic and lymphoid tissue (16). However, Wang etal. (35) classified ACAs into two groups. Group 1 consisted of those with a relatively good prognosis including those with trisomy 8, -Y and an extra copy of Ph chromosome, whereas group 2 included those with relatively poor prognosis including i(17) (q10), -7/del(7q), and 3q26.2 rearrangements. Patients in group 1 had better treatment response and survival than those in group 2. When compared to cases with no ACAs, patients with group 2 ACAs had worse survival irrelevant of the emergence phase and time. However, patients with group 1 ACAs had no adverse impact on survival when they emerged from chronic phase at the time of diagnosis. Among these 40 patients, only eight patients showed ACAs at the time of diagnosis and in the remaining ACAs that emerged during treatment. Our report confirmed that ACAs are not frequently detected in CP of CML. Out of these eight patients with occurrence of ACAs in chronic phase and at the time of diagnosis, only two progressed to blast phase and deceased. The other six are still alive. It is presumed that CML patients in whom the ACAs emerged while in chronic phase at the time of diagnosis (as in the case of these six cases) might be associated with comparatively longer progression-free survival and overall survival. This is slightly in disagreement with Jabbour etal. (36) who reported that clonal evolution or its presence at diagnosis is predictive of shortened progression-free survival and overall survival whereas its occurrence during treatment is considered as marker of treatment failure. Reports on the outcome of CP CML patients with ACAs during therapy are limited to few other studies worldwide (15, 23–25, 35, 37, 38). The frequency of clonal ACAs in the present study (15.9%) was almost consistent with the previous reports.

In the present study, hypodiploidy was associated with achievement of complete cytogenetic response. None of the patients with hypodiploid karyotypes transformed to blast phase, including patients with loss of Y chromosome which has been classified under major-route chromosomal abnormalities. This is in agreement with Bozkurt etal. (39). Transformation from chronic phase to lymphoid blast crisis phase in our study was commonly accompanied by ACAs involving either monosomy or deletion 7q. This is in agreement with a previous study by Gong etal. (23) on 2326 CML patients treated with TKIs.

The coexistence of two or more ACAs in patients with CML had been reported to have inferior survival and been categorized into a poor prognostic group (35). In the present study, it was observed that trisomy 8 was a good prognostic sign when occurring alone whereas it was detrimental when associated with other chromosome changes at initial diagnosis as described in other studies (35, 40). A few of our cases who showed complex karyotype abnormalities involving trisomy of chromosome 8, trisomy of chromosome 19, double Philadelphia chromosome, and isochromosome 17 had transformed to myeloid blast phase of CML. Gong etal. (23) observed no significant difference in median duration from emergence of ACAs to blast phase in CML patients with +8, +Ph, or other single ACAs. As a major route abnormality, trisomy 8 has been reported to confer relatively better prognosis (40), whereas minor-route ACAs with 3q26.2 rearrangement or monosomy7/7q deletion conferred a poor treatment response to TKI and dismal survival (35, 40). Gong etal. (23) reported that 3q26.2 rearrangement, i(17q), and complex ACAs were associated with a higher risk of developing myeloid blast phase, whereas monosomy 7 or deletion of 7q was associated with a higher risk of developing lymphoid blast phase. Myeloid and lymphoid blast phases were equally distributed among patients with trisomy 8 and +Ph. Three of our patients who presented with either monosomy 7 or deletion of 7q had transformed to lymphoid blast phase within 6 months from the emergence of ACAs, and two of them had poor survival rate too. This is in agreement with Gong etal. (23). Among all the 40 patients, 15 patients had transformed from chronic phase to myeloid blast crisis in a 20-month time frame. The overall survival of these patients was within 11 months after disease progressed.

Patients with isolated 3q26.2 rearrangement, -7/7q-, or i(17q) and patients with complex ACAs were reported to have a significantly shorter time interval to blastic transformation (23). In the current study, one patient showed a complex karyotype with involvement of 3q25 rearrangement, and this patient progressed to AML within a time duration of 3 months and succumbed to death soon. Out of three other patients with complex abnormalities involving iso(17q) chromosome, two patients who also had coexistence of der(22)t(9;22) had progressed to AML and had a short survival period of 34 months. One patient with i(17q) and trisomy 8 did not progress to blast crisis. The poor prognosis in i(17q) is likely to be related to *p53* deletion resulting from loss of a p arm of chromosome 17 which harbors the *p53* gene. As a tumor suppressor, *p53* plays a critical role in regulating cell-cycle arrest and apoptosis (41). The inactivation of *p53* causes genomic instability, neoplastic transformation, and disease progression. Loss of *p53* has been associated with a poor prognosis in patients with AML and MDS also (42–44). Additionally, two of our patients who had aberrations involving chromosome 15 had also transformed to AML. The abnormalities in these patients involved the long arm between 15q15 and 15q24 regions. Chromosome 15 abnormalities are identified as minor-route abnormalities (45). The tumor-suppressor gene *CCNDBP126* which is downregulated in different cancers is located on chromosome 15q. Although its role in CML is unknown, it is presumed that rearrangement involving 15q might be disrupting its function adversely (46).

Our three CML patients with ACAs consisting of complex abnormalities involving a short arm of chromosome 11 had transformed to AML. A study by Sarova etal. (47) in newly diagnosed AML reported that a complex karyotype rearrangement of chromosome 11 involved tumor-suppressor genes located at 11p15.5, such as *MIR210*, *MUC6*, *MUC2*, and *CDKNIC*. *MPPED2, DCDC5, DCDC1*, and *DNAJC24* located in the 11p14.1-p13 and 11p13 regions. There are no potential protein or RNA-coding genes at the 11p12, *NUP98* gene located at 11p15.4 which encodes for nucleoporin protein that has a role in RNAs and protein transport to the nuclear membrane. Chromosome rearrangement involving *NUP98* has also been reported to be associated with a poor outcome in *de novo* AML (48, 49). Meanwhile, the anti-apoptotic signaling pathway was reported to be involved in the rearrangement of the mixed lineage leukemia (MLL) gene on chromosome 11q23, which occurs in up to 10% of AML and was frequently associated with a poor prognosis (50). This MLL gene has been linked to a variety of translocation partners, the most common of which is *AF9* in AML (51), resulting in a variety of genetic and epigenetic abnormalities and enhanced cell survival (52). Meanwhile, MLL gene rearrangement resistance is linked to Bcl-2 family protein mutations (53).

Structural rearrangements involving chromosome 12 were also encountered in three of our CML patients with ACAs. Abnormalities comprised either translocation with another chromosome or purely deletion at breakpoint 12p12~p13. In our study, all the cases with chromosome 12 rearrangements had transformed to myeloid BP. Penas etal. (54) had reported translocation involving t(12;17)(p13;p13) in secondary AML. Chromosome 12p changes in AML, according to Walter etal. (55), have been implicated as significant adverse abnormalities similar to other complex cytogenetic abnormalities and should be considered as a high-risk cytogenetic marker for AML. Even treatment with HSCT has not been likely to change this dismal outcome, thereby warranting the need for considering other therapeutic approaches (55, 56). On chromosome 12p13 is located the *ETV6* gene which encodes a protein containing two major domains, HLH and ETS. *ETV6* has been reported to mediate carcinogenesis by various mechanisms such as activation of the kinase activity at the partner protein, modification of the original functions of a transcription factor, loss of fusion gene function, and activation of a proto-oncogene in the breakpoint of chromosome translocation (57). *ETV* is a critical transcriptional regulator involved in hematopoiesis, vascular development, and chromosomal translocations linked with hematological malignancies (58). Meanwhile, it has been reported that the *ETV6* mutations collaborate with other mutations to build their own network structure, which would stimulate the self-renewal of leukemia stem cells (59).

Additional cytogenetic alterations often appear with *ETV6* mutations, which contain various fusion genes and play a vital role in obtaining information about leukemia genesis and influencing the progression of leukemia. The rearrangement of *ETV6-LPXN* associated with progression of leukemia through regulating the microenvironment of blasts and involved in the relapse of leukemia has been reported (60). Our results prompt us to suggest that ACAs involving structural abnormalities of chromosome 12 could be used as a potential marker in predicting CML patients who are at a higher risk for disease progression and get transformed to myeloid blast phase. A recent study by Li etal. (61) on a K562 cell line demonstrated that *ETV6* regulates hemin-induced erythroid differentiation and that mechanistically *ETV6* overexpression inhibits the fibrosarcoma/mitogen-activated extracellular signal-regulated kinase/extracellular-regulated protein kinase (RAF/MEK/ERK) pathway. This finding is of great importance because the inhibitory role that ETV plays in the regulation of K562 cell erythroid differentiation *via* the RAF/MEK/ERK pathway highlights *ETV6* as a potential therapeutic target for dyserythropoiesis. Further studies are warranted to elucidate the mechanisms of *ETV6* mutations and their role in the processes of differentiation and proliferation in the early progression of leukemic alteration and whether they may also be relevant in the induction of chromosomal variability, finally prompting the development of clones with other molecular mutations and leading to myeloid blast transformation in CML patients.

Increased genetic instability and incomplete DNA repairment have been reported to generate chromosomal anomalies in CML (62). Earlier studies have clearly documented that in an already unstable genome as a result of *BCR::ABL1* in CML, the emergence of ACAs might result in further genetic instability, aberrant cellular processes, and altered metabolism (25, 62, 63). Genetic instability generates reactive oxygen species (ROS), disruption of DNA repair pathways, and inhibition of DNA damage-induced apoptosis that may result in point mutations (63). According to Bravaro et al. (63), point mutations inhibit critical contact residues between the TKIs and the target CML cells or induce a shift to a confirmation that makes TKIs unable to bind to target cells, thereby causing the development of resistance to TKIs and disease progression. Recurrent mutations and copy number alterations involving transcription factors that regulate myeloid or lymphoid differentiation might also contribute to disease progression. Additionally, in an environment of genomic instability, a number of pathways/molecules including altered DNA methylation and aberrant expression of several microRNAs (miRNAs) in association with stem cell survival and self-renewal might also be acting in concert mediating resistance to TKI therapy and subsequently disease progression. Hence, the emergence of specific ACA and the subsequent synergic activity of some or all of these cellular processes might be adversely affecting the hematologic and cytogenetic response of TKI, thereby favoring the aggressiveness of disease progression.

It is well documented that the emergence of ACAs can complicate CML pathobiology with therapy failure and disease progression as strong possibilities. In the present study, 60.6% of the Ph+ positive CML patients with ACAs showed disease progression. Among these patients, those with high-risk ACAs belonging to group 3 and group 4 showed disease progression and a reduction in overall survival. Earlier studies on ACAs in CML reported that clonal evolution was normally allied with an increased risk of hematological relapse and a decreased cytogenetic response to IM and subsequent reduction in overall survival (7, 64, 65). Our results are in agreement with the above reports, although the above studies were on clonal evolution in general and not on specific ACAs as in the present study. The present study results are also supportive of the data from the German/Swiss CML 4 trial which suggested that certain ACAs at diagnosis might identify patients at high risk of disease progression (66, 67). Despite the fact that the *BCR::ABL* gene plays a critical role in the pathogenesis of chronic-phase CML and that its sustained expression is essential for cell proliferation in the acute phase, the molecular mechanisms that lead to blast crisis are still unclear. ACAs, or molecular abnormalities, are frequent in CML cells before blast crisis (68).

Apoptosis can be considered a protective mechanism against cancer, and its disruption has significance in various malignancies. Multiple pathways lead to resistance to apoptosis in CML. Phosphorylation and activation of the PI(3)-K/Akt pathway are two primary mechanisms by which *BCR::ABL* prevents apoptosis (69, 70). PI(3) kinase activation also through protein kinase B results in the phosphorylation and inactivation of the pro-apoptotic protein BAD which is a member of the Bcl-2 family (71). BCL-2, a member of the BCL family of antiapoptotic proteins, has been found to be overexpressed in cell types harboring the BCR-ABL oncogene that contributes to reduced apoptosis (72, 73).

In chronic-phase CML, resistance to apoptosis is minimal; however, there is evidence that BCR::ABL causes resistance to apoptosis in a dose-dependent mechanism (74). A higher *BCR::ABL* mRNA expression, which is commonly linked to disease progression, could contribute to increased apoptosis to the accelerated phase and blast crisis (74, 75). Despite being a hallmark of CML, reduced apoptosis is insufficient for disease progression and it likely requires additional abnormalities such as deletion of a tumor-suppressor gene (68).

The accelerated phase and blast crises had a lower response rate than the chronic phase of CML, demonstrating the development of drug resistance *in vivo*. The presence of additional genetic aberrations that can activate the malignant clone in the absence of *BCR::ABL* is another possibility to consider (68). The resistance to therapy is caused by a variety of factors that may have an impact on disease progression. These include MDR-1 gene expression, AGP-1 expression, *BCR::ABL* reduplication or overexpression, reduced apoptosis, and possibly defective drug transport (76–78).

The p53 gene is found on chromosome 17 and has been proven to play a role in the progression of CML to blast crisis (79). Mutations, deletions, and rearrangements can cause p53 function loss, which is seen in 25% of myeloid blast crises (80, 81). In addition to loss-of-function mutations, mutant p53 proteins can function as a dominant negative isoform or combine with *BCR::ABL* to induce cell proliferation (82). The loss of function of the remaining p53 allele caused the rapid progression from chronic phase to blast crisis. Similarly, p53-deficient bone marrow cells transduced with BCR-ABL cDNA caused a fast blast crisis when transplanted (83).

Increased *BCR::ABL* mRNA and protein levels are associated with disease progression and can be caused by Philadelphia chromosome duplication, the most common cytogenetic alteration preceding disease transformation (84). The CML blast crisis has investigated into the activation of oncogenes other than the Ph chromosome. c-MYC appears to have a role in *BCR::ABL*-mediated transformation, and it could mediate its effects by either antagonizing p53’s activity or serving as a cooperative oncogene with BCR::ABL (85, 86). MYC expression is normal in chronic-phase CML; however, it is elevated in blast crisis patients (83). The overexpression of c-MYC can develop as a result of increased transcription or trisomy 8, which is common during disease progression, or polyadenylation of c-MYC m-RNA (85, 87).

In view of the latest guidelines, imatinib, dasatinib, nilotinib, and bosutinib are the FDA- and EMA-approved TKI class drugs administered as first-line standard treatment for CML (88). According to Ciftciler and Haznedaroglu (89), the choice of a primary TKI therapeutic strategy is dependent on the identification of CML patients who are at a higher risk for disease progression or TKI resistance. Therefore, they suggested that optimized integrations of the best available evidence, individual patient characteristics, physician clinical experience, and pathobiological basis are essential in order to select the best TKI for CML management. In this regard, our results prompt us to suggest that risk stratification of CML patients on the basis of the specific ACA(s) emerged could be integrated as an individual novel patient characteristic in order to select the best TKI for the treatment of CML.

However, the present study has few limitations. The results presented are from a single-center experience. Therefore, more results from the CML database have not been included. The relatively small number of patients with ACAs in the present study was also another limitation of this study: After stratifying the CML patients with ACAs into four groups, each group has a small number of patients and this has made over interpreting the analysis challenging. Hence, caution is advised in overinterpreting the data on defining the predictive significance of specific ACAs on disease progression. Further prospective studies involving a large cohort of CML patients in each group and data from the CML database are warranted to derive better significant results. In order to elucidate whether specific ACAs observed in the present study and further ACAs likely to be observed in the future might also confer useful information about disease progression and outcome, additional studies and meta-analysis across several studies would be of considerable interest.

## Conclusion

In this study on Malaysian CML patients, we demonstrated that the detection of specific ACAs and stratification of these into risk groups has a differential prognostic impact. It is reasonable to suggest that the presence of specific ACAs especially those involving complex cytogenetic abnormalities at diagnosis and during TKI treatment could be a warning sign of disease progression or treatment failure that constitutes an adverse prognostic factor in CML management. Rather than the overly simplified categorization of ACAs as major- and minor-route abnormalities, our results suggest that differential categorization based on individual ACAs could be a novel potential risk stratification system to prognosticate and guide induvial CML patients at diagnosis and during TKI treatment.

## Data availability statement

The raw data supporting the conclusions of this article will be made available by the authors, without undue reservation.

## Author contributions

RA designed the research study, interpreted and reported the cytogenetic results, corrected and revised the manuscript. AH was involved in contributing the patient samples and in the treatment of CML patients. SI, NR, and ZA collected the patient samples and carried out the conventional cytogenetic analysis. SI wrote the manuscript, NM and SS critically evaluated the manuscript. RH was involved in the haematological assessment of CML patients and AG performed the statistical analyses. All authors contributed to the article and approved the submitted version.

## Funding

This work was supported by Universiti Sains Malaysia Research University Grant (RUI) (1001/PPSP/8012243).

## Acknowledgments

The authors acknowledge the facilities extended by the Cytogenetic Laboratory Human Genome Centre, Universiti Sains Malaysia (USM), for carrying out this research work. The authors also thank the patients included in his study for their utmost cooperation.

## Conflict of Interest

The authors declare that the research was conducted in the absence of any commercial or financial relationships that could be construed as a potential conflict of interest.

## Publisher’s note

All claims expressed in this article are solely those of the authors and do not necessarily represent those of their affiliated organizations, or those of the publisher, the editors and the reviewers. Any product that may be evaluated in this article, or claim that may be made by its manufacturer, is not guaranteed or endorsed by the publisher.
